# Roux-en-Y Gastric Bypass is Associated With Increased Intestinal Glucose Uptake in Humans

**DOI:** 10.1210/jendso/bvaf172

**Published:** 2025-11-04

**Authors:** Florina Corpodean, Maryam Naseri, Michael Kachmar, Julia St Amant, Denis P Blondin, Owen T Carmichael, Vance L Albaugh

**Affiliations:** Metamor Institute, Pennington Biomedical Research Center, Baton Rouge, LA 70808, USA; Department of Surgery, Louisiana State University, New Orleans, LA 70112, USA; Biomedical Imaging Center, Pennington Biomedical Research Center, Baton Rouge, LA 70808, USA; Metamor Institute, Pennington Biomedical Research Center, Baton Rouge, LA 70808, USA; Department of Surgery, Louisiana State University, New Orleans, LA 70112, USA; Biomedical Imaging Center, Pennington Biomedical Research Center, Baton Rouge, LA 70808, USA; Department of Medicine, Centre de recherche du Centre Hospitalier Universitaire de Sherbrooke, Université de Sherbrooke, Sherbrooke, QC J1H 5N4, Canada; Biomedical Imaging Center, Pennington Biomedical Research Center, Baton Rouge, LA 70808, USA; Metamor Institute, Pennington Biomedical Research Center, Baton Rouge, LA 70808, USA; Department of Surgery, Louisiana State University, New Orleans, LA 70112, USA

**Keywords:** Roux-en-Y gastric bypass, gastric bypass, intestinal adaptation, intestinal glucose uptake, metabolic surgery, Roux limb, energy expenditure, deoxyglucose, bariatric surgery

## Abstract

**Context:**

In animal models, Roux-en-Y gastric bypass (RYGB) is associated with increased Roux limb intestinal glucose uptake that may contribute to early metabolic benefits, though prospective clinical studies are lacking.

**Objective:**

The present study aimed to test the hypothesis that Roux limb glucose uptake would increase relative to baseline in a cohort of patients undergoing RYGB.

**Methods:**

RYGB patients underwent preoperative baseline, and 3- and 6-month positron emission tomography/computed tomography postoperative imaging. Maximum and mean standardized uptake values (SUV) were measured from the following predefined regions of interest: cecum, hepatic flexure, splenic flexure, sigmoid colon, duodenal bulb, Roux limb, and common channel. SUV ratios were normalized to the spleen for assessment of longitudinal change.

**Results:**

Despite significant weight loss in all patients, no changes in Roux limb glucose uptake were observed relative to baseline; however, marked increases in glucose uptake were detected in the colon (cecum, hepatic flexure, and sigmoid colon) by 3 months that were maintained at 6 months (*P* < .05).

**Conclusion:**

RYGB is associated with increased intestinal glucose uptake in humans, but this increase appears limited to the colon in the early postoperative period up to 6 months. While the marked increases in Roux limb glucose uptake may contribute to weight loss in rodent models, this mechanism does not appear to translate to human physiology. Unexpected increases in colonic glucose uptake warrant dedicated mechanistic studies to determine the clinical significance of these changes.

Roux-en-Y gastric bypass (RYGB) is one of the most effective treatments for obesity, and also leads to impressive diabetes remission in many patients [1]. While numerous clinical trials demonstrate that RYGB is superior to other bariatric operations with respect to weight loss and diabetes remission [2, 3], the mechanisms leading to these effects remain incompletely understood. Evidence suggests that these metabolic improvements occur early (ie, days to weeks) in the postoperative course [4, 5], but dedicated studies examining the importance of these potential changes are needed.

Intestinal adaptation is one such mechanism posited to potentially drive the early and sustained improvements in insulin sensitivity and weight loss following bariatric surgery [6]. Prior studies have focused on visceral adipose tissue changes after bariatric surgery, but less emphasis has been placed on gastrointestinal changes [7]. While the physiologic stimuli are not entirely clear, surgical alteration of the gastrointestinal tract alters nutrient delivery that is hypothesized to cause structural and hormonal changes. Consistent with this hypothesis, preclinical studies demonstrate increased Roux limb mass, with corresponding increases in villus length and thickness, as well as hypertrophy of the Roux limb muscle layers [8]. This hypertrophy is associated with a doubling of L-cell number overall, but not a change in cell density [9]. These changes are not limited to RYGB but also occur after other preclinical models involving small intestinal rearrangement, like duodenojejunal bypass [10] and ileal interposition [11]. Most important, these histologic findings translate to clinical studies as well, demonstrating villus hypertrophy and biochemical changes associated with anabolic pathways detectable as early as 1 month postoperatively that are maintained up to 12 months [12, 13]. However, functional studies have not yet been conducted and whether these histologic changes equate to functional detectable changes remains unknown clinically.

In the present study, we aimed to translate preclinical findings to humans and test the hypothesis that Roux limb glucose uptake would be increased in patients following RYGB. This potential change in fuel uptake has been posited to contribute to some of the early metabolic improvements observed clinically and allow for future clinical trials and focused mechanistic studies.

## Materials and Methods

### Study Approvals and Participants

All protocols and recruitment materials were approved by the Pennington Biomedical Research Center Institutional Review Board prior to commencement of the study. Patients being evaluated for laparoscopic RYGB surgery with a body mass index (BMI) of 30 or greater were eligible for participation in this exploratory pilot and feasibility study. All participants were deemed suitable surgical candidates following initial assessment prior to being approached for study participation. Exclusion criteria were pregnancy within 6 months prior to scheduled date of surgery, diagnosis of inflammatory bowel diseases (eg, ulcerative colitis, Crohn disease), chronic kidney disease (any stage), known significant liver disease (any stage), as well as uncontrolled thyroid disease. Additionally, patients with a history of intestinal or bowel resection, or previous excessive radiation exposure or medical conditions that could interfere with positron emission tomography/computed tomography (PET/CT) scan tolerability, were excluded.

Overall, 34 patients were provided information about the study and 17 expressed an initial interest in participation. All patients were recruited and operations conducted over a 4-month period. While 12 individuals agreed to participate in the study, 2 never presented for scheduled imaging and 1 individual withdrew consent because of insufficient time to participate. Of the participants (n = 9) that entered the study, 1 participant moved more than 200 miles away prior to the 6-month PET/CT scan. Otherwise, all 8 remaining participants had 12-month follow-up as well as the planned preoperative and postoperative PET/CT imaging. Participant characteristics are presented in Table 1.

**Table 1.** Radiologic definitions of anatomical regions of interest

Region of interest	Definition
Ascending colon (cecum)	Colon segment at the inferior aspect of the cecum and tracking cephalad
Transverse colon (hepatic flexure)	Colon segment abutting the liver
Transverse colon (splenic flexure)	Colon segment abutting the spleen
Descending/Sigmoid junction (sigmoid)	Colon segment abutting the left lateral wall of the abdomen, tracked caudally
Duodenal bulb (duodenum)	First portion of the small intestine distal to the pylorus
Mid-Jejunal segment (preoperative scan)	Small bowel segment inferior to the kidneys
Roux-Limb segment (postoperative scan)	Proximal portion of the small bowel–tracked cephalad from the staple line of the jejunojejunostomy
Common channel segment (postoperative scan)	Distal portion of the small bowel–tracked caudad to the staple line of the jejunojejunostomy

All measurements of sustained uptake values (SUVs) included 10 contiguous image composites from the region of interest as defined above (3.75 mm slices per image).

All study participants underwent a baseline ^18^F-deoxyglucose (FDG)-PET scan with a corresponding low-dose CT scan of the abdomen and pelvis (PET/CT) 1 month prior to surgery before beginning any short-term low-calorie/liquid diet preoperatively. Postoperatively, participants underwent additional PET/CT scans at approximately 3 and 6 months (±10 days). Final follow-up and data collection was scheduled at 12 months, but no imaging was performed at this time.

### Positron Emission Tomography/Computed Tomography Acquisition and Quantification

Standard clinical FDG PET/CT imaging was performed using a Discovery IQ PET/CT system (GE Healthcare) at Mary Bird Perkins Cancer Center, Baton Rouge, Louisiana. The CT component was acquired with automatic tube current modulation (Auto mA) to optimize radiation dose based on patient anatomy. Participants fasted for at least 4 hours prior to PET scanning. A standard amount of 4.2 MBq of FDG tracer per kilogram of body weight was administered intravenously. This resulted in administered activity ranges of 388 to 632 MBq for the preoperative scan, 329 to 511 MBq at the 3-month follow-up, and 295 to 455 MBq at the 6-month follow-up. Following injection, participants underwent a 60-minute uptake period in a resting state.

PET imaging was then performed over a 10-minute acquisition time with a customized field of view encompassing the abdomen and pelvis. The field of view was tailored to optimize signal-to-noise ratio and ensure consistent anatomical coverage across all time points. PET images were reconstructed using the Q.Clear algorithm (GE Healthcare), which employs a Bayesian penalized likelihood approach for enhanced image quality [14].

To date, no longitudinal studies using PET/CT have measured changes in glucose tracer uptake along the intestinal tract in humans, though the technique has been validated for measuring in vivo intestinal glucose uptake [15]. For image quantification, regions of interest (ROIs) were defined during study development and prior to patient recruitment (Table 2). Definitions for ROIs were based on radiologic anatomical landmarks that could be followed serially within an individual after RYGB, including the presence of surgical staples that allow for verification of the confluence of small bowel intestinal limbs. For imaging analysis, ROIs were manually delineated on baseline and postoperative CT scans, and each scan was reviewed with its defined anatomic boundaries by the imaging technician and one of the operating surgeons (V.L.A.). These ROIs included the following: ascending colon (cecum), transverse colon (hepatic and splenic flexures), sigmoid colon, duodenal bulb, mid-jejunum (preoperative), Roux limb (postoperative), and common channel (postoperative). The spleen served as a reference region due to its relative disease invariance. Maximum and mean standardized uptake values (SUV_max_, SUV_mean_) were extracted for each ROI on corresponding PET scans, and SUV_max_ ratios (SUVRs) were calculated relative to the spleen for serial comparison. It is important to note that since the FDG tracer is administered intravenously, any tissue accumulation of tracer must result from movement of the FDG tracer from the vasculature into the intestinal tissue. Thus, for the intestinal tract ROIs, this tracer does not represent absorbed enteral glucose but rather circulating tracer from arterial blood.

**Table 2.** Patient demographics

	All
All, % (n)	100.0 (9)
Procedure, % (n)	
Initial	77.8 (7)
Conversion	22.2 (2)
Age, mean, y	35.3 ± 8.7
Sex, % (n)	
Female	100 (9)
Male	0 (0)
Race, % (n)	
White	33.3 (3)
Black or African American	66.7 (6)
Preoperative diabetes mellitus, % (n)	
Yes, insulin dependent	22.2 (2)
Yes, non–insulin dependent	22.2 (2)
No	55.6 (5)
Preoperative metformin, % (n)	
Yes	22.2 (2)
No	77.8 (7)
Preoperative GLP-1, % (n)	
Yes	22.2 (2)
No	77.8 (7)
Preoperative SGLT2 inhibitor, % (n)	
Yes	22.2 (2)
No	77.8 (7)
BMI closest to procedure, mean	47.9 ± 9.8
BMI 3 mo	39.2 ± 6.3
BMI 6 mo	35.3 ± 5.8
BMI 1 y	34.4 ± 5.9
Weight closest to procedure, mean, lbs	267.7 ± 64.1
Weight 3 mo	220.7 ± 47.8
Weight 6 mo	196.9 ± 42.1
Weight 1 y	192.7 ± 43.0
Preoperative HbA_1c_, mean, %	5.6 ± 0.6

Continuous variables are mean ± SD; categorical variables are number of observations and percentage.

**Abbreviations:** BMI, body mass index; GLP-1, glucagon-like peptide-1; HbA_1c_, glycated hemoglobin A_1c_; lbs, pounds; SGLT2, sodium glucose transporter 2.

### Roux-en-Y Gastric Bypass Operative Technique

A standard RYGB (ie, proximal gastric bypass) was performed with similar technique for all patients included in the study. Briefly, the ligament of Treitz was identified and the jejunum was divided approximately 100 cm distally from the ligament. The Roux limb was measured approximately 125 cm distally and was approximated to the biliopancreatic limb to create a common jejunojejunostomy using a surgical stapler with radiopaque staples. The Roux limb was then advanced antecolic toward the stomach. A 30-mL gastric pouch was created using a surgical stapler and then a hand-sewn, end-to-end gastrojejunostomy was created. Special care was taken to close the Petersen defect as well as the mesenteric defect at the jejunojejunostomy. Of note, there were no 30-day complications, readmissions, reinterventions, or reoperations for any of the participating patients.

### Statistical Analysis

Analyses and data visualization were conducted using R software (v4.3.1; R Foundation for Statistical Computing) and GraphPad Prism (v10.4.1). A preliminary and crude sample size calculation was conducted based on data from Franquet et al [16], a retrospective, cross-sectional imaging study that examined SUV_max_ values from intestinal tissues in patients with PET/CT at some point after gastric surgery (eg, RYGB, Billroth I or II gastrectomy). At the time of study development, no longitudinal PET/CT imaging studies examining intestinal glucose uptake had been reported and thus this pilot study was a planned exploratory study in anticipation of a future prospective clinical trial. Despite those limitations and based on the Franquet et al data, to detect a similar increase in Roux limb uptake compared to non-Roux small bowel with 80% power, estimates indicated that at least n = 5 participants would be needed in a cross-sectional study. Thus, a longitudinal design with baseline and serial PET/CT imaging would have sufficient power to detect changes in maximum Roux limb glucose uptake as well as other tissues within the abdomen. Repeated-measures mixed-effect modeling was used to assess changes in maximum and average SUVRs over time. A portion of mid-jejunum at the level of the kidneys extending inferiorly was used as a baseline for the comparisons to the Roux limb and common channel.

Normality was verified using the Shapiro-Wilk test; if the test failed for any ROI, the individual outlier data points were identified using the interquartile range rule and removed prior to analysis. Only 11 out of the total 288 tissue end points were noted to be outliers, and all of these were SUV_max_ measurements and not SUV_mean_ measurements. Post hoc Bonferroni corrections were applied for pairwise comparisons when the mixed-effects modeling indicated statistically significant differences. Given the small sample size, a nonparametric Friedman test was also conducted without outlier removal, followed by Wilcoxon signed rank tests for pairwise comparisons to confirm findings.

## Results

### Participant Characteristics

Participants characteristics (n = 9) included 7 individuals undergoing RYGB for severe obesity, while 2 others were having conversion of a previous sleeve gastrectomy to an RYGB for weight recurrence (see Table 2). All the participants were women, and most were African American/Black (62.5%). Most participants did not have diabetes and as such were not on metformin, glucagon-like peptide-1 agonists, or other medications including insulin or sodium glucose transporter 2 inhibitors. Patients with diabetes had good glycemic control, with an average glycated hemoglobin A_1c_ of 5.6%. In terms of obesity severity, the average BMI closest to the procedure was 46.7 and the cohort overall had an appropriate 1-year weight loss in which BMI decreased to 34.4.

### Positron Emission Tomography/Computed Tomography Imaging

**Figure 1.**
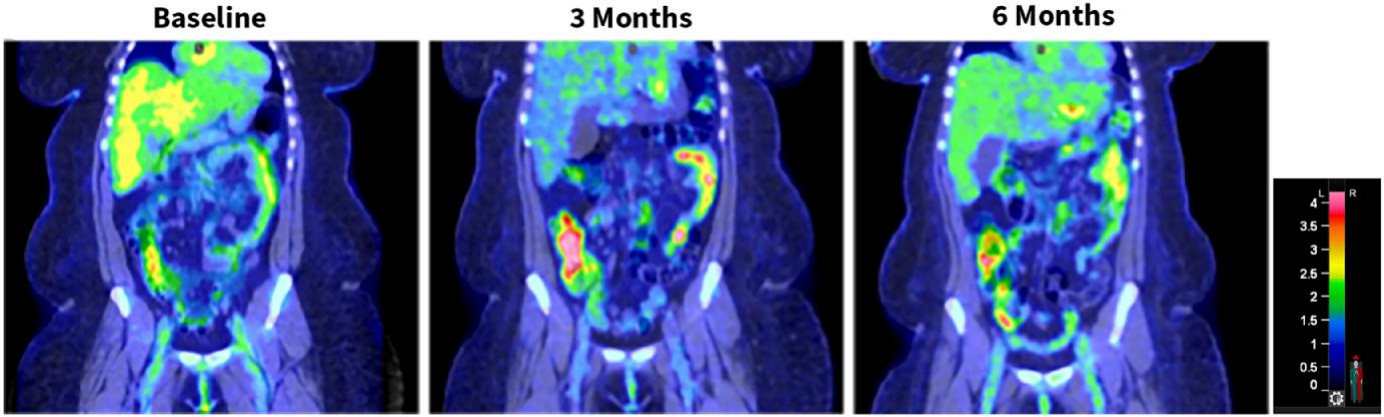
Representative coronal images of ^18^F-FDG-PET scans preoperatively (baseline) and at 3 and 6 months postoperatively. All patients underwent standard ^18^F-2-deoxyglucose-PET scans and simultaneous low-dose CT imaging of the abdomen and pelvis. Maximum and average tracer uptake was quantified from intestinal tract segments using standardized descriptions.

Representative coronal images (Fig. 1) show the extent of the standard imaging window, which included the lower portion of the chest cephalad and extended down to the mid-thigh caudally. As expected, neither maximum nor mean tracer uptake by the spleen significantly differed over time despite the marked change in body weight from baseline to 6 months (data not shown).

**Figure 2.**
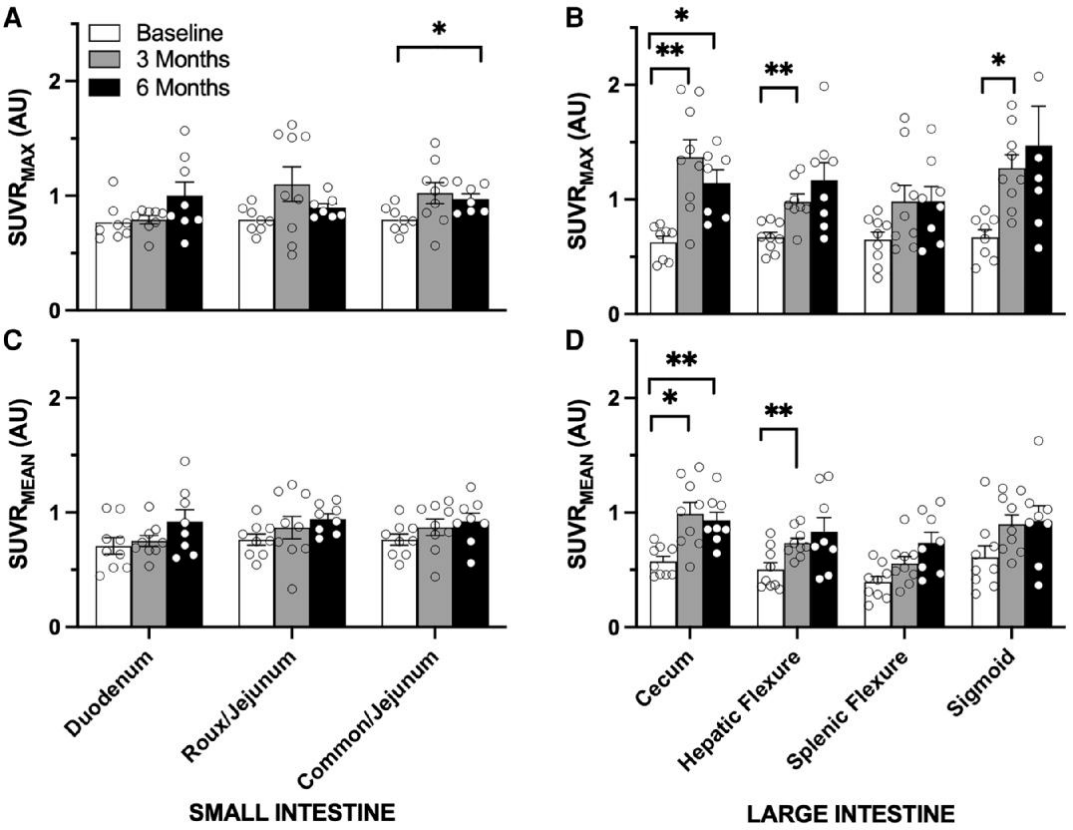
Serial maximum and mean standardized uptake values (SUV_max_) ratio (SUVR) values obtained from positron emission tomography/computed tomography (PET/CT) imaging over time. Participants underwent a standard PET/CT scan of the abdomen and pelvis preoperatively (baseline) as well as 3 and 6 months postoperatively. Maximum and mean SUVRs were measured for defined segments of A and C, the small intestine and B and D, large intestine as indicated. Individual data points are shown on top of bar plots to show individual variation in the data over time. Bar plot data represent the average SUVR_max_ and SUVR_mean_ values normalized to the spleen SUV uptake at baseline and at 3 and 6 months postoperatively as indicated on the Y-axis (n = 7-9 per tissue). *P less than .05; **P less than .01.

For most small intestine segments (Fig. 2A-2C) there were no significant increases in tracer uptake postoperatively for either the maximum or mean SUVRs. However, there was a smaller but significant increase (23%) in tracer uptake for the common channel at 6 months compared to the baseline jejunum (0.79 vs 0.97; *P* < .05).

In contrast to the small intestine, the large intestinal segments showed several robust increases in glucose tracer uptake over time that were detectable by 3 months and sustained through 6-month imaging (Fig. 2B-2D). Maximum SUVR in the cecum increased 120% at 3 months (*P* < .01) and remained elevated to 85% over baseline at 6 months (*P* < .05), without any significant difference between 3 and 6 months. Similarly, mean SUVR was also increased in the cecum, rising to 74% and 63% at the 3- and 6-month time points (*P* < .05) over baseline. More distal segments of the colon, namely the hepatic and splenic flexures, showed similar increases in maximum and mean SUVR at 3 months compared to baseline. While the hepatic flexure uptake increased approximately 46% (*P* < .01) for both maximum and mean uptake, respectively, there was no significant difference in these time points at the more distal splenic flexure. Similarly, maximum and mean uptake in the sigmoid colon also tended to be increased and sustained over time, though this was only significantly elevated for the maximum SUVR in the sigmoid colon at 3 months (91% increase compared to baseline; *P* < .05) without any detectable changes in mean SUVR.

Given the increases in colonic glucose uptake, a post hoc analysis examined whether SUVR data correlated with change in BMI over time. Of all the ROIs, the only correlations that were significant were for the splenic flexure SUVR and change in BMI at 6 months. Both Pearson (*r* = 0.75; *P* = .03) and Spearman (ρ = 0.74; *P* = .045) correlations were statistically significant, though this may be secondary to the large number of post hoc correlations performed. No other statistically significant correlations (parametric or nonparametric) were observed between change in BMI and SUVR at 3 or 6 months.

## Discussion

Even though the long-term clinical benefits of RYGB are clear, the mechanisms underlying early improvements in glycemia and weight loss are not well understood [17]. While preclinical studies [18] demonstrate direct evidence for increased intestinal glucose uptake, this mechanism has not yet been directly tested in humans. Building on this preclinical data, the present study tested the hypothesis that increased Roux limb glucose uptake would be an early effect of RYGB. Contrary to our hypothesis, while all participants experienced significant weight loss, there was minimal to no change in glucose uptake within the small intestine up to 6 months compared to baseline. In contrast, marked increases in glucose uptake were observed in the proximal colon (cecum and hepatic flexure) that were detectable by 3 months and maintained at 6 months postoperatively. These preliminary data suggest that early increases in glucose uptake are detectable following intestinal rearrangement of RYGB, but the marked changes in small intestinal glucose uptake observed in rodents may not translate clinically. It is possible that changes in glucose uptake occur within the first few days postoperatively and may have resolved by the time of the 3-month scan. Prospective clinical trials are needed to determine the magnitude and significance of these findings with respect to the metabolic improvements following RYGB.

Although the study hypothesis was not supported, imaging data revealed robust increases in glucose uptake at 3 and 6 months in the proximal colon (ie, cecum and hepatic flexure) without marked changes in small intestine. Interestingly, this is consistent with the retrospective findings of Franquet and colleagues [16], who also reported increased average values for SUV_max_ in the proximal colon (ascending colon, transverse colon) tracer uptake, but not the distal colon (descending colon, sigmoid colon). The significance of these findings is currently unknown, though it is plausible that given the significant mass of the colon these increases in SUV_max_ and average SUV represent a sizeable energy sink that could contribute to whole-body energy expenditure. For example, the response of brown adipose tissue to cold exposure is a frequently studied phenomena using PET/CT. In those studies, robust imaging changes correlate with changes in circulating metabolic fuels and energy expenditure with an approximately 45% increase over baseline for a total-body brown adipose tissue mass of 50 to 100 g [19, 20]. In the present study, the changes observed in the proximal colon are twice that of brown adipose studies (∼100% in mean SUV) and the colon has a mass that is 10-times greater than the total brown adipose mass. Prospective studies are needed to better understand the metabolic demands of these changes, which do appear to be sustained for “years” based on the prior retrospective data [16, 21].

Several studies have examined PET/CT imaging following RYGB or other foregut operations involving intestinal rearrangement or alteration of nutrient flow (eg, Billroth I or Billroth II gastrectomy, sleeve gastrectomy), though these studies are limited. In addition to the cross-sectional, case-control study by Franquet et al [16] demonstrating increased Roux limb glucose uptake, Cavin et al [21] also suggested isolated increased Roux limb glucose uptake using PET/CT in a limited patient sample (n = 3). Unfortunately, neither of these studies had preoperative data for comparison or calculation of a postoperative change in glucose uptake. A single study that included baseline imaging was able to retrospectively examine the effects of gastrectomy on glycemia in patients with advanced gastric cancer. Preoperative and postoperative PET/CT imaging demonstrated a correlation between change in fasting blood glucose with increased small intestinal PET/CT glucose measurements postoperatively, but only in individuals who had an arbitrary greater than 10-mg/dL change in postoperative fasting blood glucose that was likely affected by cachexia and/or advanced gastric cancer status [22]. Despite this evidence suggesting the possibility of a high likelihood of increased small intestinal glucose uptake following RYGB, we did not detect any changes in Roux limb glucose uptake at either 3 or 6 months compared to baseline PET/CT imaging. One of the reasons for this may be that an increase in Roux limb glucose uptake is not an early effect and takes more than 6 months to develop in humans, which would be consistent with the work by Franquet and colleagues and Cavin et al that examined patients at 13 and 7 years postoperatively, respectively [16, 21]. Regardless, the present study suggests that Roux limb glucose uptake is not driving early metabolic improvements (ie, weeks to months) postoperatively in humans.

Recent years have shown a greater focus on the intestine as a regulator of metabolism, especially with the development of incretin-based antiobesity medications. The involvement of the intestinal tract in the regulation of fasting and prandial metabolism is complex, especially with respect to obesity. How surgical changes to the intestinal tract may affect metabolism and/or insulin sensitivity is not currently well understood. Previous work has demonstrated marked differences in insulin-mediated glucose disposal between individuals with and without obesity under euglycemic-hyperinsulinemic conditions [15], but further work is needed to understand how surgical procedures and rearrangement of enteral flow affect whole-body physiology. Translational human experiments have demonstrated that experimentally altering the rate or location of nutrient flow is associated with tremendous metabolic changes even in the absence of surgery [23, 24]. Consistent with these changes in enteral nutrient flow, there are examples of increased expression of Roux limb nutrient transporters [25] as well as other adaptations in the intestine, bile acid physiology, and gut microbiota that may preclude or limit the metabolic adaptation that occurs with weight loss from dietary and lifestyle changes alone [26-28]. Further work is needed to understand how the gastrointestinal tract is altered in medical, endoscopic, and surgical obesity therapies.

Independent of surgical procedure, many other variables are affected by RYGB that could contribute to changes in intestinal glucose uptake. In particular, Honka et al [15] demonstrated that in humans with obesity compared to lean there are differences in glucose uptake within the intestine under fasting and insulin-stimulated conditions. In addition to these potential changes in tissue-specific and whole-body insulin sensitivity [4, 29-31], decreased caloric intake with altered macronutrient consumption, shifts in the gut microbiota and circulating bile acids [28, 32], as well as potential differences in the catabolic/weight loss response postoperatively could be contributing to changes in intestinal glucose uptake. In the present study, imaging was performed under standard clinical conditions (ie, fasting), making changes in insulin-dependent glucose uptake undetectable. These potential insulin-dependent changes within the intestine and others should be examined in future studies. Moreover, it is important to note that at the time of the baseline PET/CT, patients likely did not have the same degree of caloric restriction as after surgery. In this study it is tempting to speculate, but any or all of these variables could have underlying effects on intestinal glucose uptake. Thus, further studies are needed to understand how these variables might affect intestinal glucose uptake. Regardless, serial PET/CT is a feasible and promising tool to better understand how fuel uptake changes in various physiologic states and how that may be altered by metabolic surgery.

The present study has several strengths, most notably the longitudinal design and ROIs that were defined by the imaging and surgical teams prior to study enrollment. Most important, these ROI definitions were based on fixed, anatomic landmarks that do not significantly change postoperatively and are easily identifiable (ie, radiopaque staples) at crucial landmarks that can be tracked over time. Moreover, all image analysis was completed by the imaging technician and one of the operating surgeons who jointly reviewed all scans to verify anatomic landmarks and promote reproducibility.

While the present study is the first longitudinal study attempting to quantify intestinal glucose uptake over time following RYGB, the study is not without limitations that may limit generalizability. Most notable is the pilot nature of the protocol as well as the small sample size based on retrospective data. Second, 2 of the 9 patients studied underwent conversion of a prior sleeve gastrectomy to an RYGB, and potential differences in these related but distinct procedures cannot be resolved. These patients were recruited as part of the pilot, anticipating a robust increase in Roux limb glucose uptake following primary RYGB that may differ in patients undergoing conversion from sleeve compared to primary RYGB. Regardless, the exclusion of those individuals does not change the interpretation of the imaging findings, as these individuals grossly responded similar to the individuals undergoing primary RYGB. Given the small sample size, we may have missed evolving changes that would be detectable with a larger group, specifically for the slight, nonsignificant trends in small intestine glucose uptake. Additionally, all participants were female and any sex-specific effects are unknown. While this is not ideal, our clinical practice is approximately 85% female, which is similar to other bariatric and weight loss studies. Also, effects of diabetes or preoperative/postoperative dietary changes (eg, prolonged fasting, low-calorie diets) that could alter intestinal glucose uptake cannot be ruled out. Postoperatively, these patients are asked to consume approximately 60 to 80 g of protein per day, which could also be associated with changes in intestinal and microbiome metabolism. Regardless, the potential effects of these factors must be studied and accounted for in future prospective trials using data from this cohort for power calculations. Finally, despite prior data suggesting effects of RYGB on intestinal glucose uptake, the present study focused on early (<6 months postoperatively) and may have missed changes that develop as part of chronic changes related to intestinal adaptation. Future studies should focus on patients further from surgery to understand how changes in intestinal fuel uptake might be contributing to weight loss and weight maintenance.

## Conclusion

The use of routine PET/CT imaging to measure serial intestinal glucose uptake in patients following RYGB is feasible. While data did not support the hypothesis that Roux limb glucose uptake would be increased by 3 or 6 months postoperatively, it did reveal unexpected and robust increases in glucose uptake by the proximal colon. The clinical significance of these findings is currently unknown but could be related to alteration in various factors including gut microbial changes, bile acid metabolism, and resolution of severe obesity. Future clinical studies are necessary to better understand the changes in intestinal fuel uptake and their potential effect on the early metabolic changes following RYGB or other bariatric operations, as well as antiobesity pharmacotherapy.

## Data Availability

Data sharing is not applicable to this article as no datasets were generated or analyzed during the current study. Analytic methods will be made available to other researchers on reasonable request. Due to the small sample size, data and study materials will not be made publicly available to maintain patient confidentiality.

## Abbreviations

BMI: body mass index
FDG: ^18^F-deoxyglucose
PET/CT: positron emission tomography/computed tomography
ROI: region of interest
RYGB: Roux-en-Y gastric bypass
SUVs: standardized uptake values
SUV_max_: maximum standardized uptake value
SUV_mean_: mean standardized uptake value
SUVR: SUV_max_ ratio
